# Compound hemizygous variants in *SERPINA7* gene cause thyroxine‐binding globulin deficiency

**DOI:** 10.1002/mgg3.1571

**Published:** 2021-02-07

**Authors:** Yanlan Fang, Hong Chen, Qingqing Chen, Chunlin Wang, Li Liang

**Affiliations:** ^1^ Pediatric Department The 1st Affiliated Hospital of Zhejiang University School of Medicine Hangzhou China

**Keywords:** compound variants, *SERPINA7* gene, single‐nucleotide variants, thyroxine‐binding globulin deficiency

## Abstract

**Sub‐heading:**

Compound hemizygous variants in SERPINA7 gene.

**Background:**

Thyroxine‐binding globulin (TBG) is encoded by *SERPINA7* (OMIM. 314200) which is located on Xq22.3. *SERPINA7* variants caused TBG deficiency which does not require treatment, but the decreased thyroxine may be misdiagnosed as hypothyroidism. We discovered some variants of TBG caused by alterations that differ from previously reported.

**Materials and Methods:**

In this study, we enrolled 32 subjects from 10 families and sequenced the *SERPINA7* genes of TBG‐deficient subjects. Then, variants were analyzed to assess their effect on TBG expression and secretion. Bioinformatics database, protein structure, and dynamics simulation were used to evaluate the deleterious effects. Finally, we identified 2 novel and 4 known variants, and found 26 of 30 subjects carried the p.L303F. The DynaMut predictions indicated the variants (p.E91K, p.I92T, p.R294C, and p.L303F) exhibited decreased stability.

**Conclusion:**

Analyses revealed the p.L303F change the protein stability and flexibility, and it had an impact on the function of TBG, but when coexisted with other variants it might change the conformational structure of the protein and aggravate the damage to the protein. We speculated that the existence of a higher number of variants resulted in lower TBG secretion.

## Graphical abstract

The p.L303F that previouly identified as a variant had an impact on the function and stability of TBG, and when coexisted with other variants it might change the conformational structure of the protein and aggravate the damage to the protein. The existence of a higher number of variants resulted in lower TBG secretion.

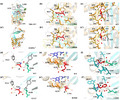

## 1 INTRODUCTION

In humans, thyroxine‐binding globulin (TBG) is one of the major serum transport proteins of thyroid hormones (THs), and the other major transport proteins include transthyretins and human serum albumin. Although high amounts of albumin are found in serum, TBG has a greater affinity for thyroxine (T4). Abnormalities in the functionality and amount of TBG cause alterations in the total T4 amount in serum but not the level of free T4. TBG deficiency, including complete TBG deficiency (TBG‐CD) and partial TBG deficiency (TBG‐PD), manifests as a normal metabolic state but is diagnosed by an abnormal result from a thyroid function test called euthyroid hypothyroidism. Physicians may not recognize these deficiencies and provide an inappropriate prescription, which would lead to side effects.

As far as we know, all inherited TBG‐PD variants that have been reported to date are caused by missense variants (Pappa et al., 2015), and this type is the most common form of inherited TBG deficiency, with a frequency of 1 in 4,000 newborns. Since the first description of the human *SERPINA7* gene in 1986, a series of variants and single nucleotide polymorphisms (SNPs) have been identified (Flink et al., 1986). The pre‐TBG protein is 415 amino acids in length and contains a 20‐amino acid signal peptide that directs it into the endoplasmic reticulum. Among these variants, p.L303F, corresponding to p.L283F in the mature protein is the most common SNP. Although we have previously referred to the p.L303F (rs1804495) as p.L283F or TBG‐poly, we will refer to this variant as p.L303F, since this is its standard name in the Human Gene Mutation Database (http://www.hgmd.cf.ac.uk/). This SNP is often associated with other variants in the *SERPINA7* gene, whose role is not yet defined (Bertenshaw et al.,; Janssen et al., 2000; Mannavola et al., 2006; Mori et al., 1990; Su et al., 2003). Although p.L303F was previously considered to be a “benign” variant, two recent reports have shown that the serum TBG levels were lower in males with p.L303F variant compared to the normal controls (Chen et al., 2020; Dang et al., 2019). The mean TBG concentration in heterozygous females with TBG‐PD is usually higher than half the normal level, and this level is intermediate between those found in affected and unaffected males. Affected males present with the classic TBG deficiency, which includes low total T4 and T3 levels, low but detectable TBG expression, and a normal free TH level in serum. The clinical phenotype is often mistaken for hypothyroidism and leads to unnecessary treatment or even drug‐induced hyperthyroidism.

In this study, the clinical features and laboratory results from 10 families with TBG deficiency were described. Gene sequencing revealed six variants in the *SERPINA7* gene and five compound hemizygous variants involving these six variants. The effects of TBG‐Poly on thyroid function were assessed in individuals by independent t‐tests. To further confirm the pathogenicity of these *SERPINA7* gene variants, we assessed the expression level and conformational dynamics of these single and compound variants through in vitro assays.

## 2 PATIENTS AND METHODS

### 2.1 Subjects

The study was reviewed and approved by the Ethics Committee of The First Affiliated Hospital, Zhejiang University, China (No. 2017‐863), and was conducted in agreement with the Declaration of Helsinki Principles. Written informed consent was obtained from the patients’ parents.

### 2.2 Clinical evaluations

We evaluated the probands and their parents for clinical manifestations and laboratory thyroid function tests including TT3, TT4, FT3, FT4, TSH, and TBG.

### 2.3 Thyroid function tests

The concentrations of TBG were determined using a chemiluminescent immunometric assay (Siemens Healthcare Diagnostics Products Limited) with an automated analyzer. Serum thyroid stimulating hormone (TSH) was assayed by chemiluminescence method. Thyroid hormones are measured by automated immunoassay platform. All the samples from each individual were measured in the same assay run.

### 2.4 Genetic diagnosis

Genomic DNA was extracted from the peripheral blood samples of the probands and their parents. The sequence variants detected in the *SERPINA7* gene were described according to the NCBI entry NM_000354.6 (NC_000023.11). The description of variants was according to the Human Genome Variation Society (HGVS) sequence variant nomenclature(Dunnen et al., 2016). The correct nomenclature for variants were checked applying Mutalyzer (https://mutalyzer.nl/).

### 2.5 Analysis of the conservation and pathogenicity of the variants

A variety of vertebrate TBG protein sequences were downloaded from UniProt (https://www.uniprot.org/), and Clustalx 2.1 software was used for sequence alignment. The sequence alignment results were displayed using the online ConSurf Server (http://consurf.tau.ac.il/).

The pathogenicity and conservation of the variants were analyzed using the webserver PREDICT‐SNP (https://loschmidt.chemi.muni.cz/predictsnp1/). This tool provides the consensus of several different predictors (SIFT, SNAP, PolyPhen‐2, MAPP, and PredictSNP) accompanied by a confidence score for the predictions(Bendl et al., 2014).

The changes in the dynamics and stability of five TBG variants were predicted using the DynaMut server (http://biosig.unimelb.edu.au/dynamut/prediction). Specifically, using this server, a normal mode analysis (NMA) of WT TBG using two different tools, Bio3D (Grant et al., 2006) and ENCoM (Frappier et al., 2014), and the effects of every single variant on the TBG dynamics and stability induced by changes in vibrational entropy were then assessed. The output was presented in terms of the predicted changes in stability (in kcal/mol) and entropy energy (in kcal/mol/K) between the WT and variant structures.

### 2.6 Molecular modeling

The DynaMut server (http://biosig.unimelb.edu.au/dynamut/prediction) was used to predict the interactions among amino acid residues, and the results were displayed using PyMOL software. The three‐dimensional structure of human TBG (PDB accession code 2RIW and 4X30) was used to study the effect of the identified variants on the protein conformation (Qi et al., 2011). The structural representation was generated using the molecular visualization system in the open‐source foundation (PyMOL 2.4, https://pymol.org/2/). An image of the variant p.Ile310Phefs* was generated using Swiss‐Model (https://swissmodel.expasy.org/interactive).

### 2.7 Construction of *SERPINA7* variants

The wild‐type (WT) full‐length human *SERPINA7* cDNA (GenBank: M14091.1) with EcoR1 and BamH1 restriction enzyme sites was chemically synthesized (Tsingke, China) and cloned into the plasmid vector pcDNA3.1 (+). As described previously, the variants and a 15–20‐bp terminal homologous sequence were introduced into the vector. The circular plasmid was complementarily paired (Table S1), and *Escherichia coli* DH5α was used for positive plasmid amplification (Chen et al., 2019; Gibson et al., 2010). Alleles carrying two or more mutations were constructed in the same way.

### 2.8 Transient transfection of *SERPINA7* cDNA

We included 15 groups in the analysis: negative control (only pcDNA3.1(+)), positive control (pcDNA3.1(+)‐Wild type), and 13 experimental groups (pcDNA3.1(+)‐mut, where mut refers to c.271G>A, c.275T>C, c.631G>A, c.880C>T, c.909G>T, c.927del, c.[271G>A;909G>T], c.[275T>C;909G>T], c.[631G>A;909G>T], c.[880C>T;909G>T], c.[909G>T;927del], c.[271G>A;631G>A], and c.[271G>A; 631G>A;909G>T]). For transient transfection, HEK‐293T cells were cultured in 24‐well plates until they reached 60%–70% confluence, and the cells were then transfected with 500 ng of plasmid using the Lipofectamine™ 3000 transfection reagent (Thermo Fisher Scientific).

### 2.9 Quantitative real‐time PCR

Forty‐eight hours after transfection, total RNA was isolated according to a previously described method (Liu et al., 2336T>C). cDNA was obtained via reverse transcription using a reverse transcription kit (TaKaRa). The sequences of the forward and reverse primers used for PCR amplification were 5′ATCTGGTTCTCTTGGTACTTG3′ and 5′TGGATGACATCTTGTAGAGAGTGGC3′, respectively.

### 2.10 Detection of TBG secretion from cells by enzyme‐linked immunosorbent assay (ELISA)

Forty‐eight hours after transfection, the TBG level in the cell supernatant was determined using an ELISA kit (*Boster*), which use the sandwich format and biotin–streptavidin chemistry. The captured antibody was diluted to a final concentration of 1–10 μg/ml in bicarbonate/carbonate antigen‐coating buffer, and 100 μL of the diluted antibody was pipetted into each well of a microtiter plate. The plate was covered with adhesive plastic and incubated at 37°C for 30 min. The coating solution was removed, and the plate was washed three times with PBS buffer. Subsequently, 200 μl of blocking buffer was pipetted into each well to block the residual protein‐binding sites. The plate was covered with adhesive plastic and incubated for 1.5 h at 37°C. One hundred microliters of the diluted antibody was then pipetted into the wells with the control, standard, and diluted samples, and 100 μl of the diluted ABC solution was then pipetted into these wells. The absorbance of each well at 450 nm was read using an ELISA reader within 15 min after addition of the stop solution.

### 2.11 Western blot

Forty‐eight hours after transfection, the proteins of the cells were extracted using a Whole Cell Extraction Kit (Abcam). The samples were analyzed by 12% SDS–PAGE and immunoblotted as described in the section on immunoblot analyses. Western blotting was performed using a polyclonal anti‐TBG antibody (Abcam), and the immunoreactive bands on the membrane were visualized using enhanced chemiluminescence reagents (Super Signal, Pierce) and a CLINX ChemiScope and ECL system (CWBIO) (Liu et al., 2336T>C).

### 2.12 Visualization of the allele frequency

Data on *SERPINA7* variant allele frequencies were downloaded from the Genome Aggregation Database (gnomAD) (http://gnomad.broadinstitute.org/). The latest version of gnomAD was downloaded (https://gnomad.broadinstitute.org/downloads), and data on variants of the TBG protein and their corresponding allele frequencies were obtained. In addition, data on the Chinese population were obtained from the Chinese Millionome Database (CMDB) (https://db.cngb.org).

### 2.13 Statistical analysis

GraphPad Prism 7 and Fiji ImageJ for Mac were used for the statistical analyses.

The independent samples t‐test was used for comparisons between two groups. All in vitro data were analyzed using at least three independent replications. The data are presented as the means ± SDs (*n* ≥ 3).

## 3 RESULTS

### 3.1 Clinical evaluations

Our first diagnosis of TBG deficiency was an 11‐year‐old boy and his identical twin brother (family E, II‐1 and II‐2) who were transferred from a clinic to our hospital due to thyroid dysfunction. Eight months ago, they were admitted to another hospital because they were dissatisfied with their height. Since the thyroid function examination of the proband showed low TT4 and normal TT3 and TSH, it was diagnosed as hypothyroidism at the beginning. Patients were given 75 μg/day of sodium thyroxine, which increased to 125 μg/day after 8 months until drug‐induced hyperthyroidism appeared. Laboratory tests revealed a persistent decrease in TSH. Further tests revealed that they had subnormal serum levels of TBG. After discontinuation of thyroxine therapy, hyperthyroidism disappeared and TSH returned to normal in both patients. These clinical features support the diagnosis of TBG‐PD and iatrogenic hyperthyroidism (Table S2).

In the following 2016–2018, we collected 10 more TBG‐deficient families in clinical practice. At first, these patients came to our hospital because of short stature. On examination their TT4 and/or TT3 levels were found to be abnormal, but FT3, FT4, and TSH were normal. Due to previous misdiagnosis, we further assessed the serum TBG levels in these patients. All of them had lower than normal serum TBG levels. Among them, the serum TBG levels of the four probands (II‐1 of family C, II‐1 of family D, II‐1 of family H, and II‐1 of family J) were lower than the measurable levels (<3.5 μg/ml) and were determined to be TBG‐CD. The remaining six probands had lower TBG levels than normal and were identified as TBG‐PD (3.5–13.0 μg/ml). And, five parents (I‐1 of family A, I‐2 of family C, I‐1 of family F, I‐1 of family G, and I‐2 of family H) were also identified as TBG‐PD. All the probands and their parents had normal FT3, FT4, and TSH levels. The laboratory results of these probands and their parents are summarized in Table 1.

**Table 1:** Thyroid function tests and TBG concentration in all probands and their parents

Subject	Family members	Variants in TBG	TT3 (nmol/L)	TT4 (nmol/L)	FT3 (pmol/L)	FT4 (pmol/L)	TSH (mU/L)	TBG (μg/ml)
A	Proband (II‐1)	p.L303F Hemizygotes	1.32	**53.4↓**	6.13	13.8	2.70	**9.8↓**
	Mother (I‐2)	p.L303F Heterozygotes	1.82	65.9	5.73	14.2	2.33	20.3
	Father (I‐1)	p.L303F Hemizygotes	1.13	**52.4↓**	5.82	12.7	2.45	**8.8↓**
B	Proband (II‐1)	p.L303F Hemizygotes	2.11	62.8	7.49	16.0	0.41	**10.1↓**
	Mother (I‐2)	p.L303F Heterozygotes	1.98	72.9	4.89	19.7	3.19	19.8
	Father (I‐1)	Wild type	2.56	73.9	5.27	20.8	3.12	16.5
C	Proband (II‐1)	p.I92T and p.L303F Hemizygotes	1.04	**43.4↓**	6.10	22.8	2.97	**<3.5↓**
	Mother (I‐2)	p.I92T and p.L303F Heterozygotes	2.01	55.6	4.35	17.8	2.30	**11.6↓**
	Father (I‐1)	p.L303F Hemizygotes	1.89	55.8	5.16	19.8	3.01	14.6
D	Proband (II‐1)	p.I92T and p.L303F Hemizygotes	1.35	**30.6↓**	7.37	22.7	2.38	**<3.5↓**
	Mother (I‐2)	ND	1.89	69.0	3.46	18.9	1.78	20.2
	Father (I‐1)	Wild type	2.02	67.5	4.58	20.3	1.47	25.3
E	Proband (II‐1)	p.A211T and p.L303F Hemizygotes	1.27	**41.5↓**	5.64	17.27	1.31	**11.0↓**
	Twin brother (II‐2)	p.A211T and p.L303F Hemizygotes	1.12	**35.4↓**	5.69	15.41	0.96	**10.4↓**
	Litter brother (II‐3)	p.L303F Hemizygotes	1.20	100.8	4.67	18.50	1.47	13.0
	Mother (I‐2)	p.L303F Heterozygotes	1.18	105.2	4.43	18.04	1.47	21.0
	Father (I‐1)	p.L303F Hemizygotes	1.38	91.1	4.89	19.06	1.50	13.8
F	Proband (II‐1)	p.A211T and p.L303F Hemizygotes	1.32	**46.7↓**	5.95	14.5	2.19	**12.6↓**
	Mother (I‐2)	p.A211T Heterozygotes and p.L303F Homozygotes	1.45	60.3	5.29	15.3	2.18	17.7
	Father (I‐1)	L303F Hemizygotes	1.10	55.6	4.10	12.4	2.04	**12.2↓**
G	Proband (II‐1)	p.A211T Heterozygotes and p.L303F Homozygotes	1.79	**55.2↓**	6.25	11.91	2.78	14.2
	Mother (I‐2)	p.L303F Heterozygotes	1.33	89.7	5.22	17.18	2.09	18.2
	Father (I‐1)	p.A211T and p.L303F Hemizygotes	1.24	57.7	4.36	19.57	3.58	**12.7↓**
H	Proband (II‐1)	p.E91K, p.A211T, and p.L303F Hemizygotes	1.10	**36.9↓**	6.18	18.7	1.84	**<3.5↓**
	Mother (I‐2)	p.E91K, p.A211T, and p.L303F Heterozygotes	1.03	**52.6↓**	3.14	12.8	2.28	**10.9↓**
	Father (I‐1)	Wild type	1.50	67.9	5.58	17.4	2.30	21.9
I	Proband (II‐1)	p.R294C and p.L303F Hemizygotes	1.24	**49.9↓**	5.71	18.6	2.03	**6.59↓**
	Mother (I‐2)	p.R294C Heterozygotes and p.L303F Homozygotes	1.13	**52.8↓**	5.76	13.8	2.15	13.5
	Father (I‐1)	p.L303F Hemizygotes	1.19	**53.1↓**	4.38	16.7	2.40	14.5
J	Proband (II‐1)	p.I310Ffs* and p.L303F Hemizygotes	**0.72↓**	**31.7↓**	4.82	24.0	2.27	**<3.5↓**
	Mother (I‐2)	ND	1.84	66.7	4.21	17.8	2.13	19.6
	Father (I‐1)	Wild type	1.97	70.8	3.84	16.9	1.97	22.3

Note

TT3: total triiodothyronine (1.02–2.96 nmol/L); TT4: total thyroxine (55.4–161.3 nmol/L); FT3: free triiodothyronine (2.77–6.31 pmol/L); FT4: free thyroxine (10.5–24.4 pmol/L); TSH: thyroid stimulating hormone (0.3 to −4.34 mIU/L); and TBG: thyroxine‐binding globulin (13.0–39.0 μg/mL).

A‐I: Nine families; ND: Not done; The abnormal values are in bold.

### 3.2 Genetic diagnosis

**FIGURE 1:**
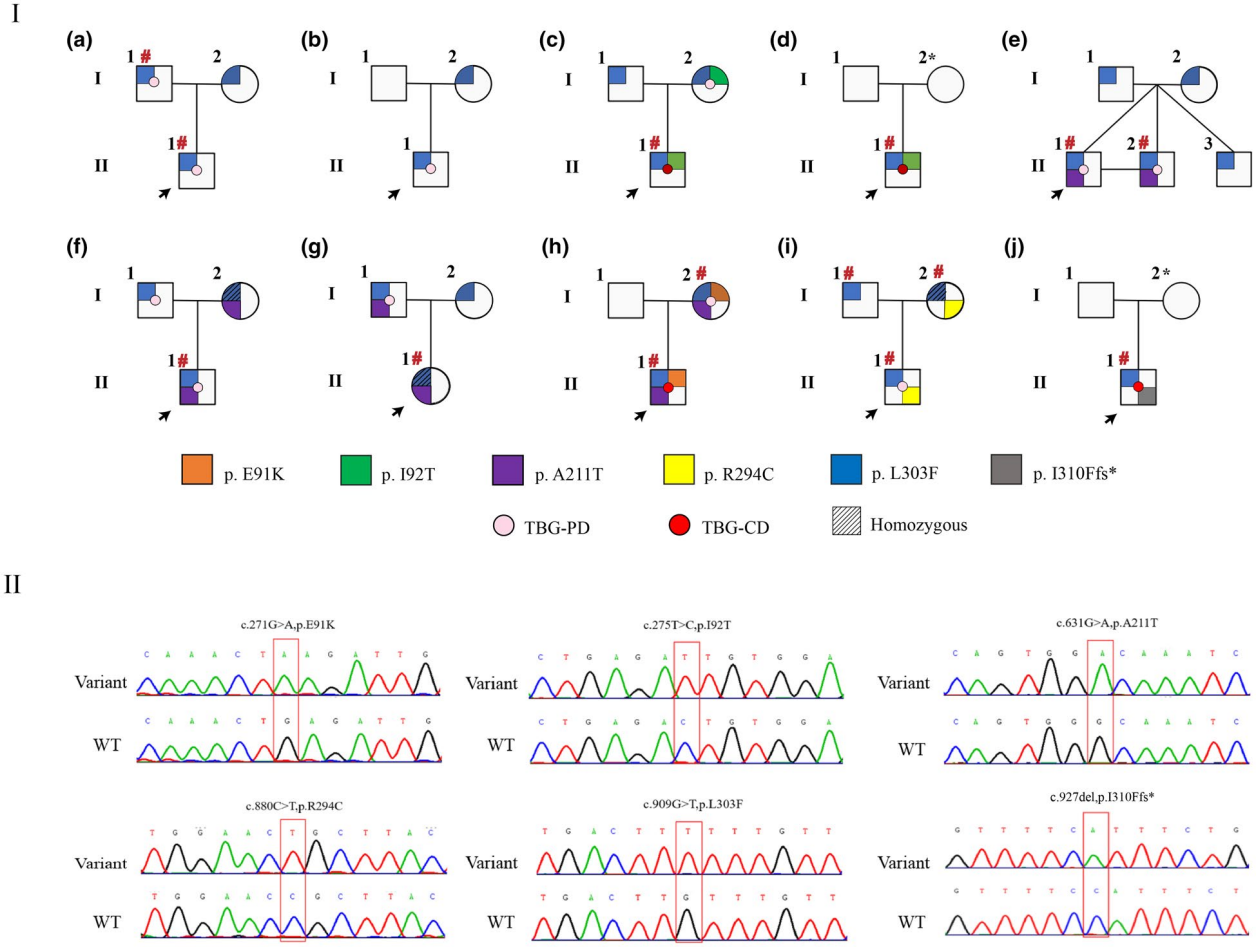
Ten pedigrees with TBG deficiency and Sanger sequencing chromatograms. I: A‐J indicates 10 pedigrees with TBG deficiency. The arrowheads denote the probands. The affected individuals with partial TBG deficiency are shown as pink dots in the middle of the box. The affected individuals with complete TBG deficiency are shown as red dots in the middle of the box. The orange square shows that the clinically affected subject harbors the p.E91K variant; the green square shows that the clinically affected subject harbors the p.I92T variant; the purple square shows that the clinically affected subject harbors the p.A211T variant; the yellow square shows that the clinically affected subject harbors the p.R294C variant; the blue square shows that the clinically affected subject harbors the p.L303F variant; the gray square shows that the clinically affected subject harbors the p.I310Ffs* variant; and the unfilled symbols indicate that that the subjects are clinically unaffected. The asterisks indicate the individuals who are not available for gene sequencing. Line filling indicates homozygosity. # indicates individuals with reduced total thyroxine levels. II: Sanger sequencing chromatograms. The variants are indicated by an arrow

Sanger sequencing revealed six variants in the *SERPINA7* gene: c.271G>A/p. E91K, c.275T>C/p.I92T, c.631G>A/p.A211T, c.880C>T/p.R294C, c.909G>T/p.L303F, and c.927del/p.I310Ffs*. All the patients, including those with TBG‐PD and TBG‐CD, carried compound hemizygous variants (c.[275T>C;909G>T], c.[631G>A;909G>T], c.[880C>T;909G>T], c.[927del;909G>T], and c.[271G>A;631G>A;909G>T]), with the exception of four, who carried only one SNP (c.909G>T). Notably, two novel variants, c.271G>A/p.E91K and c.927del/p.I310Ffs*, were identified (Figure 1). Among the 10 families, 86.7% (26/30) individuals carried p.L303F variant. All patients (18/18) with abnormal thyroid function results had the p.L303F variant.

### 3.3 Analysis of the conservation and pathogenicity of the variants

**FIGURE 2:**
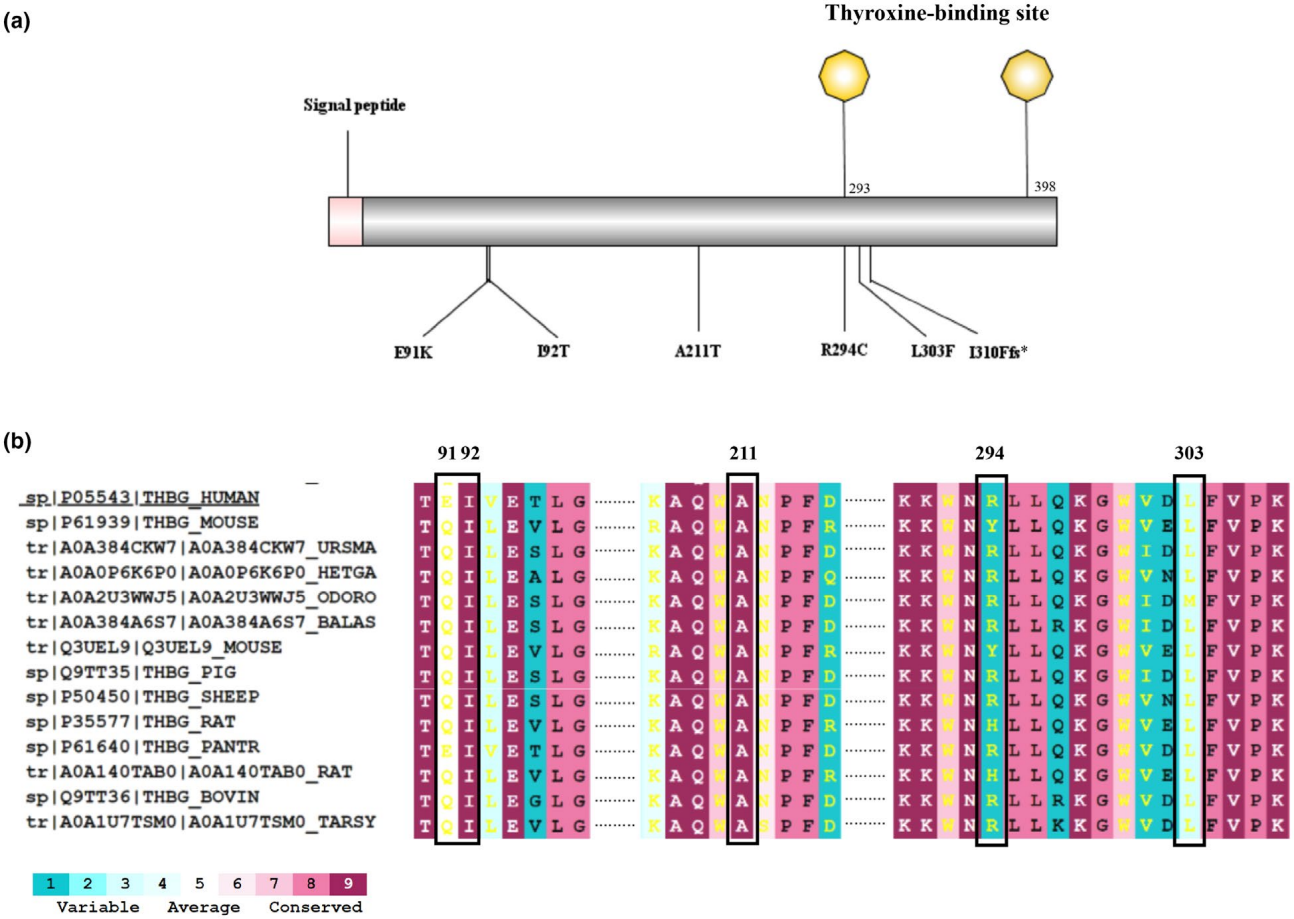
Analyze distribution and conservation of TBG variants. (a) Schematic of human preproTBG protein showing the location of 6 different TBG missense variants, and the resulting amino acid changes. (b): Conservation of p.E91K, p.A211T, p.R294C, and p.L303F in TBG proteins from 14 different vertebrates

Immature TBG has a putative signal peptide (20‐amino acid) at the N‐terminus. Figure 2a shows the location of these six variants in the immature TBG. The conservation analysis showed that the WT residues at I92 and R294, but not those at E91, A211, and L303, were conserved in vertebrates (Figure 2b).

The novel missense variant p.E91K was predicted to be deleterious by four of five predictors with high confidence scores of 0.61, 0.50, 0.53, and 0.80. And the variants p.I92T, p.R294C, and p.L303F were found to be pathogenic by all five prediction software programs. In contrast, the predictions for variant p.A211T indicated that it is likely benign (Table 2).

**Table 2:** Predictions and confidence scores for the TBG variants obtained with the PREDICT‐SNP server

Variants	Reference ID	PredictSNP		MAPP		PolyPhen−2		SIFT		PhD‐NAP	
		Prediction	Accuracy	Prediction	Confidence score	Prediction	Confidence score	Prediction	Confidence score	Prediction	Confidence score
p.E91K	—	Deleterious	61%	Neutral	64%	Deleterious	50%	Deleterious	53%	Deleterious	88%
p.I92T	—	Deleterious	79%	Deleterious	81%	Deleterious	55%	Deleterious	79%	Deleterious	77%
p.A211T	rs2234036	Neutral	83%	Neutral	63%	Neutral	68%	Neutral	76%	Neutral	72%
p.R294C	rs182605024	Deleterious	55%	Deleterious	46%	Deleterious	59%	Deleterious	45%	Deleterious	46%
p.L303F	rs1804495	Deleterious	72%	Deleterious	57%	Deleterious	43%	Deleterious	53%	Deleterious	57%

To study these changes in the structural stability of TBG induced by the five variants, we utilized the DynaMut webserver, which predicts the variant‐induced changes in the dynamics and stability of a protein. The DynaMut predictions showed that the p.E91K, p.I92T, and p.L303F variants destabilized the protein and that the p.I92T variant resulted in the lowest stability. In contrast, the p.A211T and p.R294C variants appeared to be relatively stable (Table 3).

**Table 3:** Predicted results obtained with the DynaMut server after the amino acid change(s) in the TBG variants

Variants	ΔΔG			ΔΔS_vib_
	DynaMut	ENCoM	DUET	ENCoM
p.E91K	−0.082 kcal/mol (Destabilizing)	−0.067 kcal/mol (Destabilizing)	−0.327 kcal/mol (Destabilizing)	0.084 kcal.mol^−1^.K^−1^ (Increased molecular flexibility)
p.I92T	−2.727 kcal/mol (Destabilizing)	−0.603 kcal/mol (Destabilizing)	−3.066 kcal/mol (Destabilizing)	0.754 kcal.mol^−1^.K^−1^ (Increased molecular flexibility)
p.A211T	0.618 kcal/mol (Stabilizing)	0.177 kcal/mol (Destabilizing)	−0.637 kcal/mol (Destabilizing)	−0.221 kcal.mol^−1^.K^−1^ (Decreased molecular flexibility)
p.R294C	0.152 kcal/mol (Stabilizing)	0.032 kcal/mol (Destabilizing)	−0.135 kcal/mol (Destabilizing)	−0.040 kcal.mol^−1^.K^−1^ (Decreased molecular flexibility)
p.L303F	−0.716 kcal/mol (Destabilizing)	0.584 kcal/mol (Stabilizing)	−1.750 kcal/mol (Destabilizing)	−0.730 kcal.mol^−1^.K^−1^ (Decreased molecular flexibility)

### 3.4 Three‐dimensional structure modeling

**FIGURE 3:**
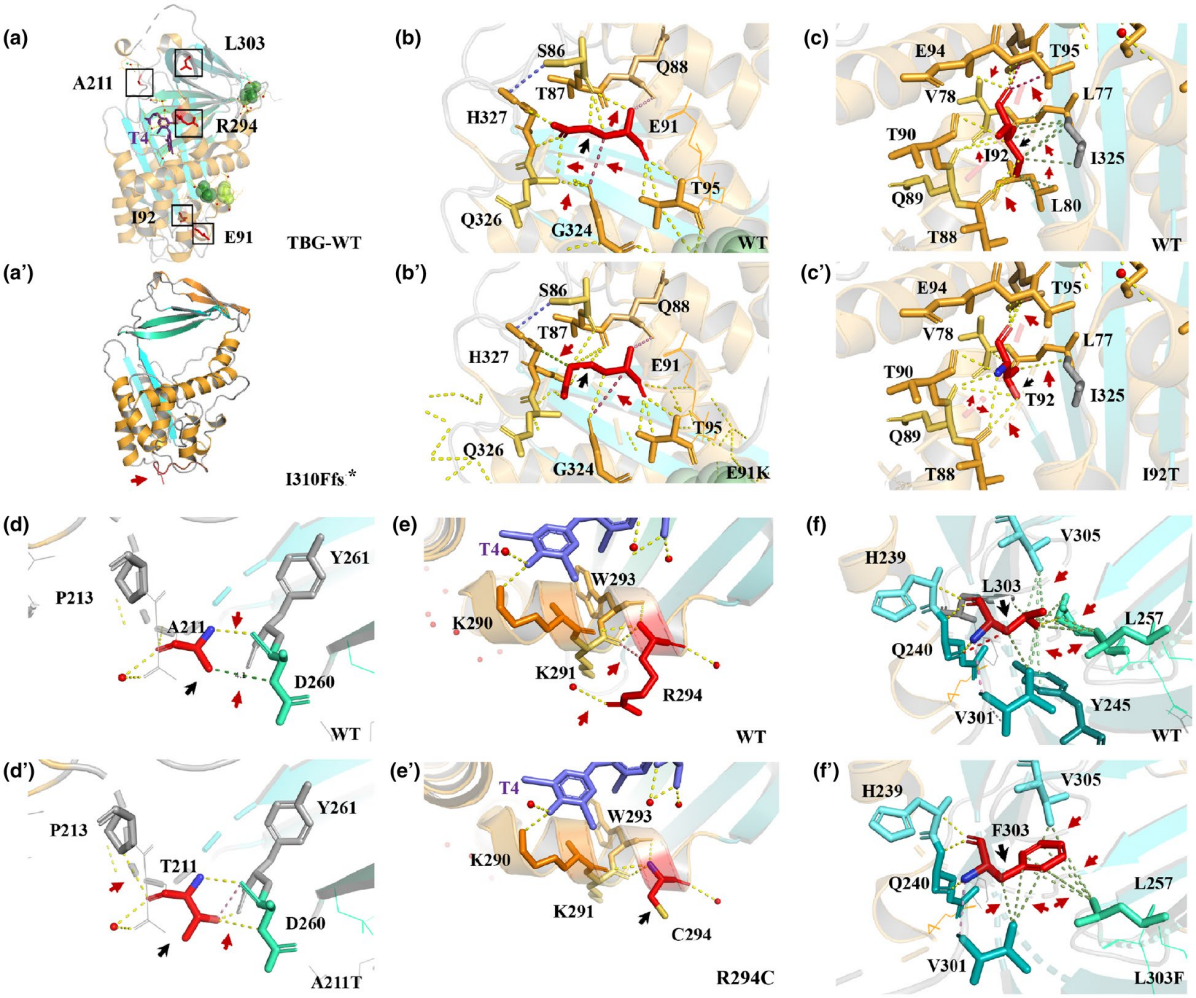
Prediction of interactions between amino acid residues. The WT and variant residues are colored in red and are represented as sticks alongside the surrounding residues that are involved in any type of interactions. The red ball indicates H_2_O. The red arrows point to interactions between the altered amino acids, and the black arrows point to WT or variant amino acid residues. The colors of the contacts are based on the following: yellow, hydrogen bonds; red, ionic interactions; green, hydrophobic contacts; and pink, carbonyl contacts. (A) Three‐dimensional structure of the WT TBG. Black boxes mark the locations of the five variants. (A′) Three‐dimensional structure of the p.I310Ffs* variant. A red arrow points to the truncated protein terminus. (B and B′) Interaction between the local amino acids of the WT and the p.E91K variants. (C and C′) Interaction between the local amino acids of the WT and the p.I92T variants. (D and D′) Interaction between the local amino acids of the WT and the p.A211T variants. (E and E′) Interaction between the local amino acids of the WT and the p.R294C variant. (F and F′) Interaction between the local amino acids of the WT and the p.L303F variants

All structural images and amino acid interactions were drawn using PyMOL. The novel frameshift variant c.927del causes early translation termination and thus produces a truncated TBG protein (Figure 3A′). The variant p.E91K, which involves the mutation of a glutamic acid residue to lysine, formed a hydrogen bond with S86, T95, Q326, and H327. Due to the size difference between glutamic acid and lysine residues, the residue in the variant is not located in the correct position to produce the same hydrogen bonds as the WT residue (Figure 3B,B′). The differences between residues I92 and T92 might interfere with the core structure of the domain: the I92 residue is buried in the core of a domain, whereas the variant residue is markedly smaller, which might create a void in the core of the protein. Moreover, as shown in Figure 3C,C′, the p.I92T variant led to a loss of hydrophobic interactions in the protein core, which appears to imply that these variants exhibit more severe pathogenicity. The analysis of the variant p.A211T revealed that the residue in the variant interferes with its interactions with other molecules or parts of the protein because the threonine residue is larger than the alanine residue. In contrast, due to the different hydrophobicity of alanine and threonine, the p.A211T mutation might cause a loss of some of the hydrophobic interactions with other molecules on the protein surface (Figure 3D,D′). Differences in charge and hydrophobicity between arginine and cysteine explain why p.R294C results in a loss of interaction with other molecules. Moreover, residues in the vicinity of the mutated residue are annotated as part of a binding site of TBG. The variant can affect the local structure and thus the TBG‐binding site (Figure 3E,E′). Further analysis showed that p.L303F, which is buried in the core of the domain, is smaller than F303, and the F303 residue might thus interfere with the core structure of the domain (Figure 3F,F′).

### 3.5 Decreased levels of TBG were associated with p.L303F variant

**FIGURE 4:**
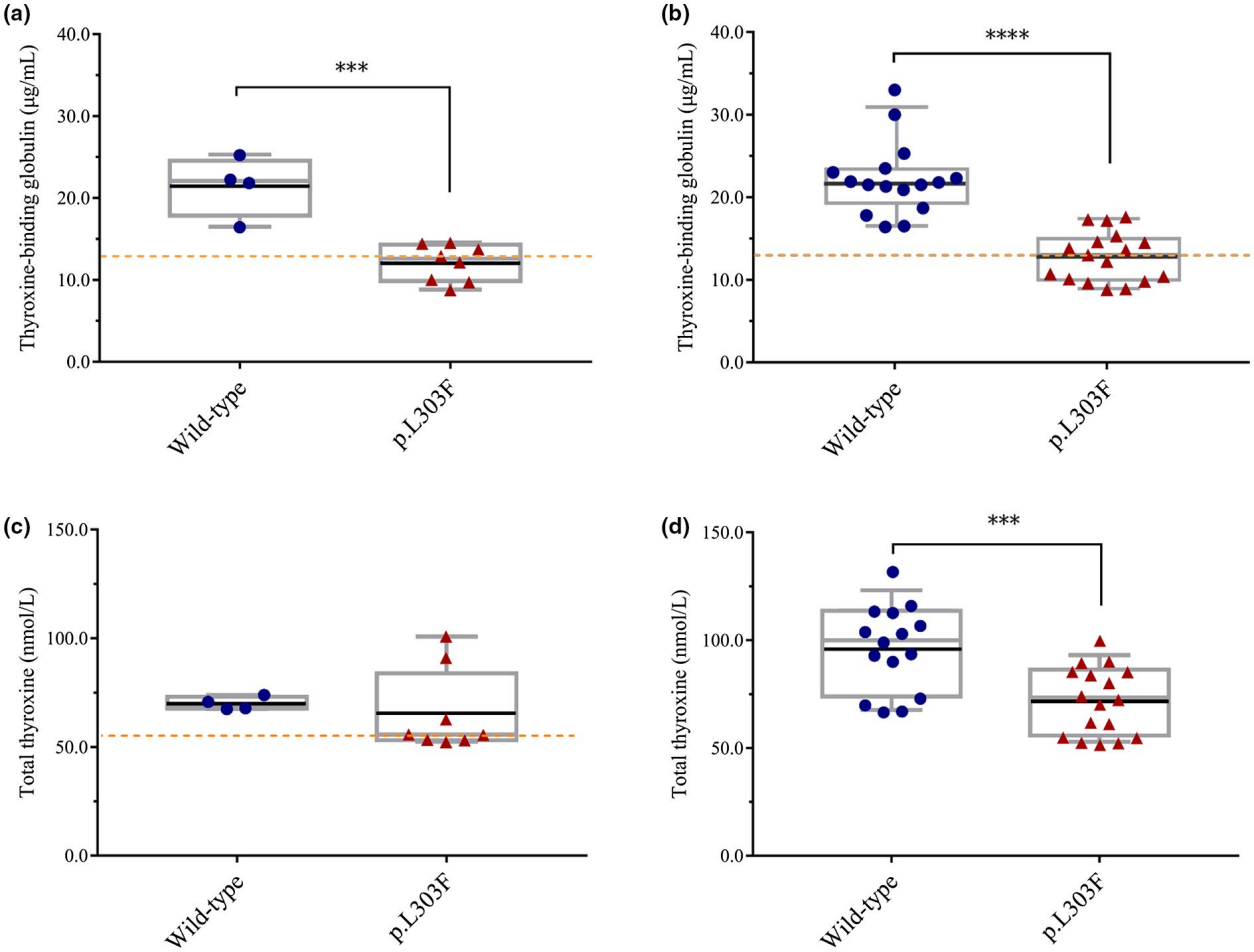
Box plots of pairwise comparisons for TBG and TT4 concentrations. The black horizontal lines represent the mean, and boxes represent the quartiles of data range including the median. The scatter points show all data points. (a) The comparison of TBG levels between two groups in this study. (b) Our research data incorporates previous research data of TBG levels for two sets of analysis. Group Wild type: WT individuals with *SERPINA7* gene sequencing. (c) The comparison of TT4 levels between two groups in this study (d) our research data incorporates previous research data of TT4 levels for two sets of analysis. Group p.L303F: individuals with p.L303F hemizygotes or p.L303F homozygotes. The orange dotted line indicates threshold of the normal TBG level and TBG deficiency clinically (13.0 μg/ml). ***p < 0.001 and ****p < 0.0001

To determine whether the p.L303F variant affects serum TBG levels, we compared the serum TBG levels of subjects with p.L303F hemizygous or homozygous variants to WT subjects. As shown in Figure 4a, the TBG levels of p.L303F group(12.1 ± 0.7983, *n* = 8)was significantly lower than that of the WT group (21.5 ± 1.831, *n* = 4) (*p* = 0.0002). To test the reliability of our results, we first compared our data with that reported in the literature (Chen et al., 2020; Dang et al., 2019; Mannavola et al., 2006).

PubMed articles on TBG deficiency as of June 2020 are collected. The p.L303F variant is a common SNP locus, which is carried at a higher rate in the population. Moreover, we considered that this variant may affect the level of TBG in individual serum. In addition, the control group should be WT individuals with *SERPINA7* gene sequencing. Males with only the p.L303F hemizygous variant and females with only the p.L303F homozygous variant were screened. Meanwhile, serum TBG levels of WT individuals with *SERPINA7* gene sequencing were collected as controls. After these articles are included, relevant data are collected from each paper. Nevertheless, there are few studies and reports on TBG levels of individuals with p.L303F variants. Only three previous studies offer eligible individual data. A total of 15 WT individuals and 15 hemizygous males and 2 homozygous females were collected including our study. Raw data of serum TBG level in individuals are shown in Table S3. As shown in Figure 4b, the serum TBG level of p.L303F variant (12.79 ± 0.7301, n = 17) was significantly lower than wild type (21.69 ± 1.038, n = 15), *p* < 0.0001. There were no significant differences between groups in the level of TT4 (*p* = 0.664) most likely due to the low sample size of this study (Figure 4c). We pooled *SERPINA7* p.L303F variant and/or WT samples along with samples harboring this variant and/or wild type from previous research (Chen et al., 2020; Dang et al., 2019; Mannavola et al., 2006). The TT4 levels of p.L303F group were significantly lower than the WT group (*p* = 0.0006; Table S3, Figure 4d).

### 3.6 Analysis levels of secreted TBG and intracellular TBG

**FIGURE 5:**
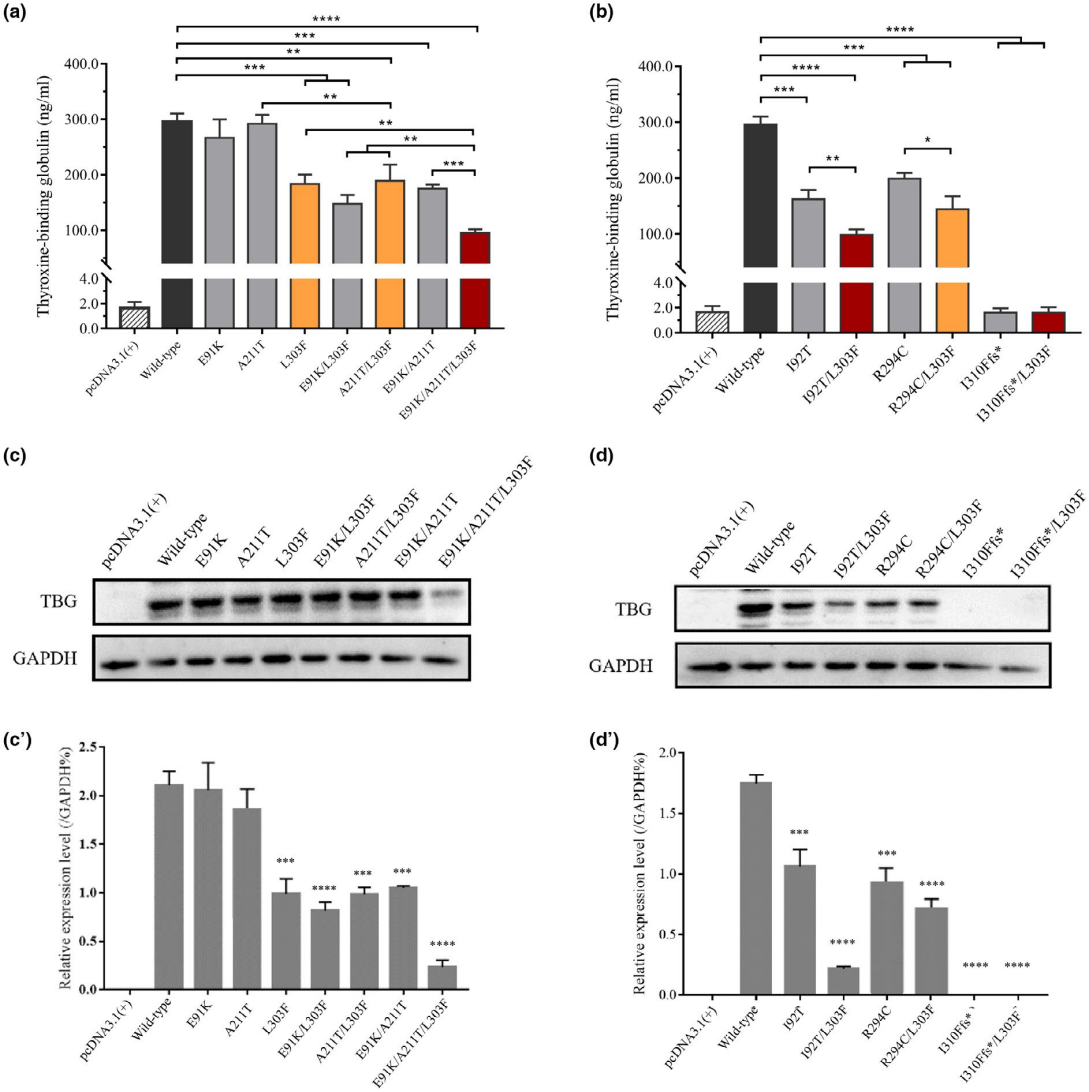
Extracellular and intracellular TBG levels. (a and b) Detection of extracellular TBG secretion by ELISA. The genotypes of the TBG‐CD patients are filled with red, the genotypes of the TBG‐PD patients are filled with orange, and the wild type is filled with black color. (c and d) The protein expression level was measured by Western blot analysis. The data are presented as the means ±SDs, *p < 0.05, **p < 0.01, ***p < 0.001, and ****p < 0.0001

The in vitro expression of TBG was analyzed by ELISA and Western blot analysis. Quantitative PCR results revealed no *SERPINA7* gene differences in mRNA levels among groups (Figure S1). The exocrine levels of different variants of TBG were measured by ELISA, and the results showed that the single variants p.E91K‐ and p.A211T‐induced TBG secretion, similar to the results obtained with the wild type. The levels of TBG with the p.I92T, p.R294C, and p.L303F variants were approximately 54.77%, 67.25%, and 63.13% of the WT levels, respectively. The analysis of the compound hemizygous variants showed that the TBG secretion levels obtained with the p.E91K/L303F, p.A211T/L303F, and p.R294C/L303F variants were 50.04%, 63.90%, and 67.26% of the WT levels, respectively. In addition, the p.I92T/L303F and p.E91K/R294C/L303F variants resulted in secretion levels that were 33.28% and 32.25% of the WT levels, respectively. As expected, TBG secretion could not be detected with the deletion variant (Figure 5a,c).

We then further examined the effects of the variants on the intracellular TBG protein level through a Western blot analysis (Figure 5b,d). A Western blot analysis using anti‐TBG as the antibody showed that the expression level of TBG protein with the p.E91K variant was similar to the WT level. Decreased protein expression levels were obtained for the other variants, particularly the p.I92T/p.L303F and p.E91K/p.A211T/p.L303F variants. The protein band of the p.I310Ffs* variant was not detected for imaging. The results from the Western blot analysis were consistent with the ELISA results, which indicated that the identified variants affect the stability, but not the extracellular secretion, of TBG.

### 3.7 Allele frequency analysis

**FIGURE 6:**
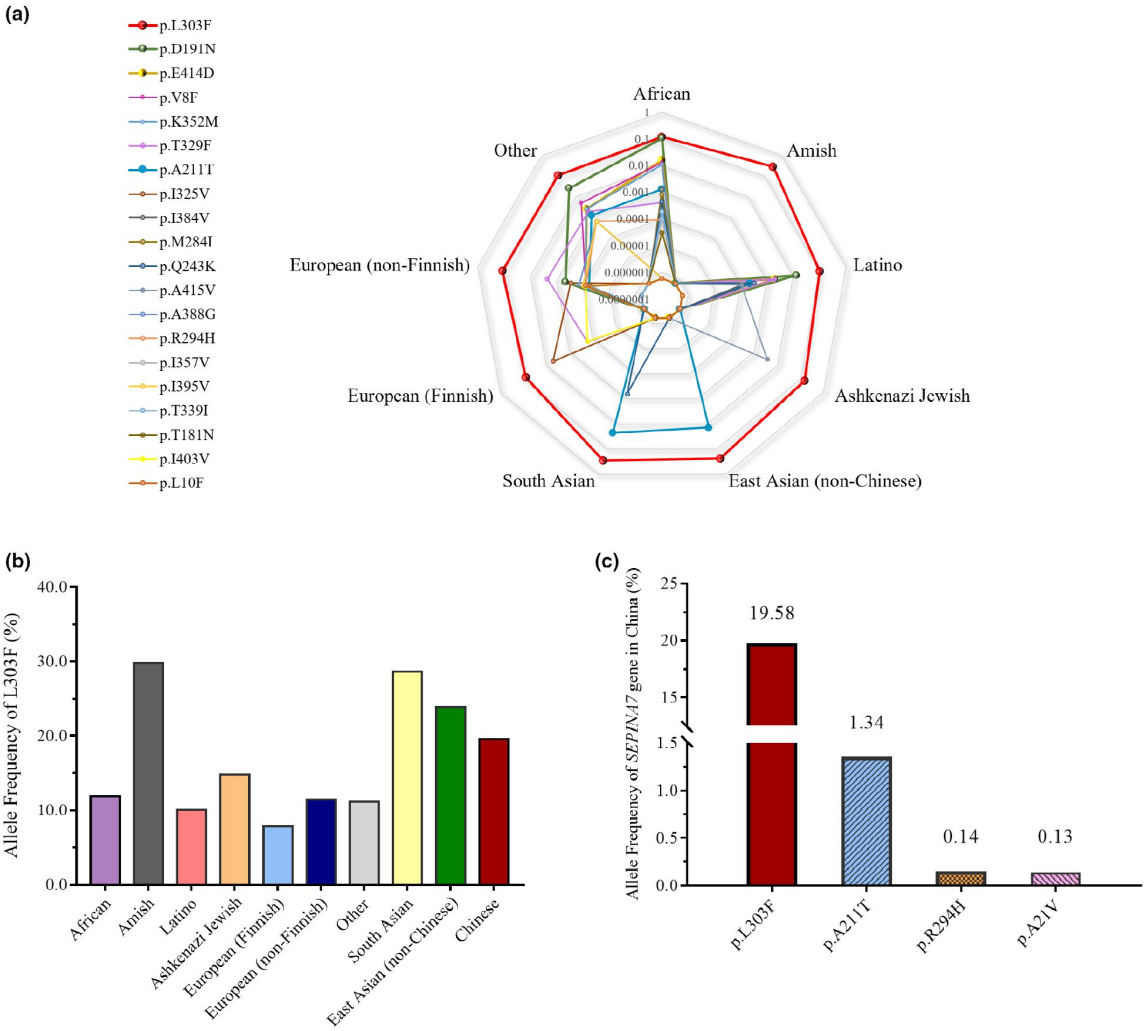
Frequency distribution of the *SERPINA7* gene variants in the African, Amish, Latino, Ashkenazi Jewish, East Asian, Finnish, non‐Finnish European, South Asian, China, and other populations. (a) Frequency distribution of the top 20 variants in the *SERPINA7* gene among different populations detailed in the Genome Aggregation Database. (b) Frequency distribution of the p.L303F variants among different populations. (c) Allele frequency of the p.A21V, p.A211T, p.R294C, and p.L303F variants in Chinese

The frequency and distribution of *SERPINA7* variants in the Chinese Millionome database and gnomAD are summarized in Figure 6. The p.L303F variant is the most common in the general population. The p.A211T variant is found at a higher frequency only in the East Asian (non‐Chinese) (2.3%) and South Asian (1.4%) populations (Figure 6a). The p.R294C and p.I92T variants, which are found only in the non‐Finnish European population, are extremely infrequent and are rare variants. There are some differences in distribution of p.L303F variant in different regions, being lowest in European. The highest frequency is found in the Amish (29.8%), followed by the South Asian (28.6%) and East Asian (non‐Chinese) populations (23.9%), whereas the Finnish European (7.8%) and Latino (10%) populations carry relatively low frequencies. This allele has a population frequency of 13.88%, which is the highest frequency of *SERPINA7* gene. There are some differences in distribution of p.L303F variant in different regions (Figure 6b). The highest frequency of p.L303F variant was observed in Amish (29.79%), followed by South Asians (28.62%) and East Asians (non‐Chinese) (23.87%). According to the Chinese Millionome database, the mutation frequency of p.L303F variant in Chinese was 19.58%. In addition, only four variants, p.A21V, p.A211T, p.R294C, and p.L303F, were identified among the Chinese population, as detailed in the Millionome database. Similar to the results in other populations, p.L303F is the most common variant in the Chinese population, with a frequency of approximately 19.6%. In contrast, the frequency of the p.A211T allele was found to equal 1.34%, which is similar to that reported in East Asia (1.4%) and less than that found in South Asia (2.26%). No such gene has been reported among the Amish, Ashkenazi Jew, and European populations. The p.I92T and p.R294C alleles have not been reported in the Chinese population (Figure 6c).

## 4 DISCUSSION

Herein, we present 32 subjects from 10 families. Most of these proband came to the hospital for further examination due to their short stature and found the TT4 reduction by accident. Our first case (II‐1 and II‐2 of family E) was misdiagnosed as hypothyroidism and treated with levothyroxine until iatrogenic hyperthyroidism occurred. TBG is rarely tested in pediatric clinic because it is rarely used as an indicator of disease diagnosis and treatment. Thyroid hormones play a key role in the growth and development of children. Therefore, some children of short stature with decreased serum TT4 are easy to be misdiagnosed as thyroid insufficiency and receive unnecessary treatment.

Among the 10 families, 86.7% (26/30) individuals carried p.L303F variant. Interestingly, all affected individuals (18/18) carried p.L303F variant, also known as TBG‐poly. The TBG‐poly had been considered as a benign variant in early studies, but our conclusion seems inconsistent with the previous literature (Janssen et al., 1992). The statistical results of the serum TBG levels in the p.L303F hemizygotes were significantly lower than WT individuals (*p* = 0.0002) in our subjects, which is consistent with two recent reports (Chen et al., 2020; Dang et al., 2019). The TBG levels of >13 μg/ml has been described as a cutoff between normal and abnormal. From Figure 4a,b, it is clearly shown that all data points are distributed near the critical value line and are significantly different from the TBG level of WT individuals. Although TT4 levels in p.L303F hemizygotes (median = 55.7 nmol/L) were lower than those in WT individuals (median = 70.0 nmol/L) in our subjects, there was no significant difference between the two groups due to our insufficient sample size. However, when we combined the subjects’ samples with the previous study samples, TT4 levels in the p.L303F group were significantly different from those in the WT group (*p* = 0.0006).

We found that most of the patients carry X‐linked TBG deficiency‐causing variants that are not considered classical variants but are rather present in a new form as compound single‐nucleotide variants. Of note, we purport that our analysis involved the use of a variety of predictive methods for determination of the pathogenicity of each variant and their association with TBG deficiency, and the results suggest that these five missense variants appear to be related to TBG deficiency. The predictions obtained with the DynaMut server revealed that the single variants p.E91K, p.I92T, and p.L303F reduced the stability of the protein and that the variants p.A211T and p.R294C exerted minor if any effects on the stability. Four probands were diagnosed with TBG‐CD in our study, and one of the probands has a frameshift variant in amino acid residue 310 and a stop codon in amino acid residue 320, which results in a truncated protein and can be considered a de novo variant. Another two probands from different families have the same variants, p.I92T and p.L303F. Both algorithms predicted that p.I92T is likely a pathogenic variation. And, the comparison of TBG expression among p.I92T, p.L303F, and p.I92T/L303F revealed that the lowest level was obtained with the p.I92T/L303F variant, which can explain the sociological results. The proband from family H, which carries all three variants, presented with TBG‐CD, whereas the proband from family E, F, and G carries p.A211T/L303F and has less severe TBG deficiency. These results showed that the compound variants resulted in a significant decrease in TBG secretion. The presence of a higher number of variants resulted in a greater effect on TBG secretion, which might be a superposition effect. As mentioned above, a western blot analysis showed that the observed alterations in intracellular TBG protein expression observed in the variant and WT groups were similar to the serum levels of TBG measured by ELISA, which indicates that these variants alter the stability of TBG in cells without altering the exocrine capacity of this protein.

In summary, we identified six variants of *SERPINA7* and five compound variants that had not previously been reported as a cause of TBG deficiency. Endocrinologists should better identify inherited TBG deficiency to help prevent misdiagnosis and unnecessary treatment of the disease. More importantly with respect to the etiology of TBG deficiency, the findings identified a de novo mechanism: compound variants in *SERPINA7* might lead to conformational changes in the structure and ultimately influence the stability of the protein. Unlike previous studies, our results indicate that the p.L303F variant changes the function of TBG. And, when it coexists with other variations may exacerbate the damage to the function of TBG. The combination of a higher number of variants resulted in decreased TBG secretion.

Some limitation of this study must be acknowledged. First, we did not further examine the T4‐binding ability of these variants. Second, despite our best efforts, we have only a handful of individual clinical data. For such a high allele frequency, these data are far from sufficient. In the future, more clinical data and functional evidence will be needed for better analysis.

## CONFLICTS OF INTEREST

The authors declare no conflict of interest.

## AUTHORS CONTRIBUTIONS

CL Wang and L Liang conceived and designed the research. YL Fang, H Chen, and QQ Chen performed the experiments and analyzed the data. YL Fang, H Chen, QQ Chen, CL Wang, and.

L Liang wrote the manuscript.

## Supporting information

Fig S1

Table S1‐S3

## Data Availability

All data generated or analyzed during this study are included in this article.
