# Supplementary figures and images for: Compound hemizygous variants in SERPINA7 gene cause thyroxine‐binding globulin deficiency
Source: Mol Genet Genomic Med. 2021 Feb 7;9(2):e1571. doi: 10.1002/mgg3.1571 (PMC8077092; doi:10.1002/mgg3.1571)

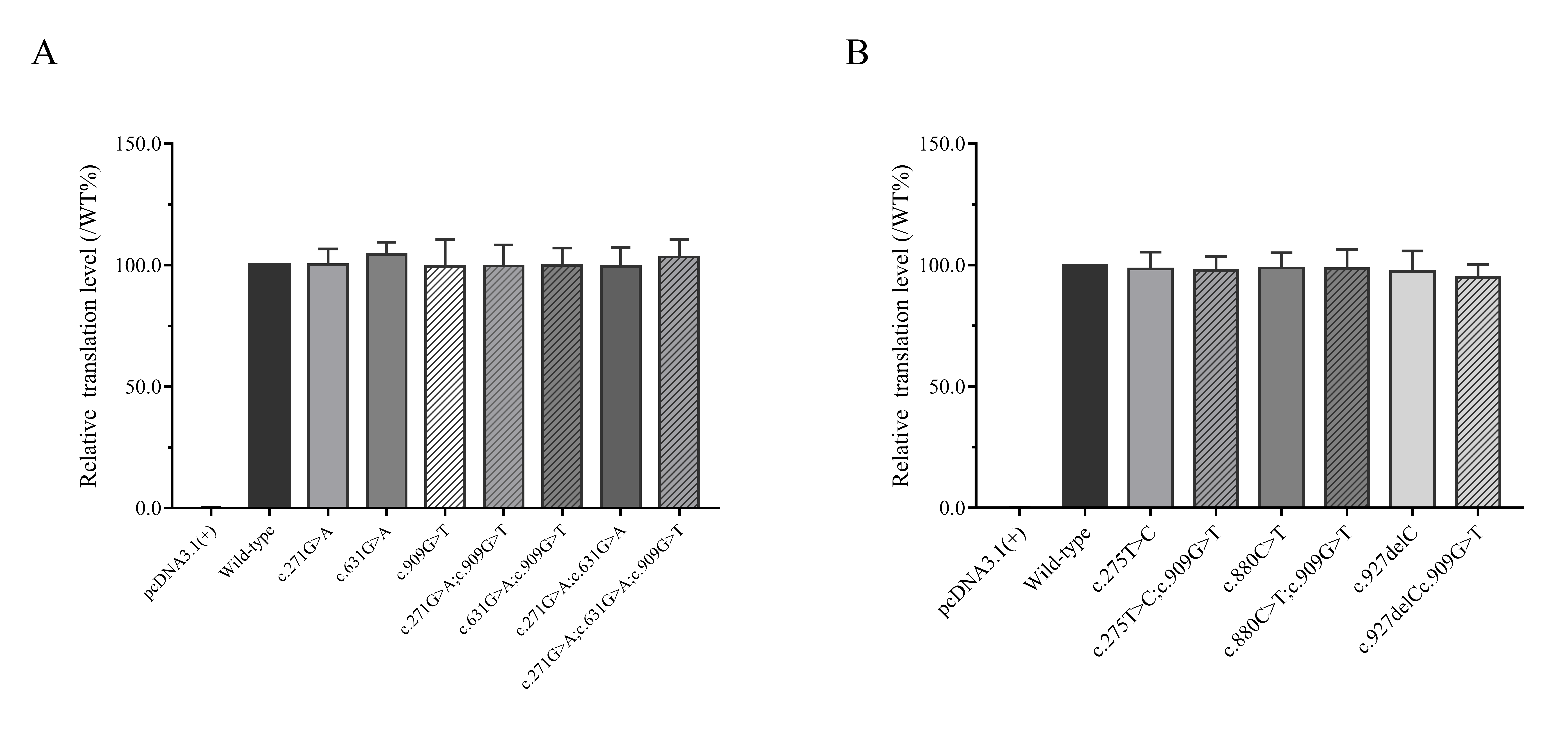

Supplement: Supplementary file 1 — Fig S1 [file MGG3-9-e1571-s001.tif]
