# Supplementary material for: Compound hemizygous variants in SERPINA7 gene cause thyroxine‐binding globulin deficiency
Source: Mol Genet Genomic Med. 2021 Feb 7;9(2):e1571. doi: 10.1002/mgg3.1571 (PMC8077092; doi:10.1002/mgg3.1571)
Supplement: Supplementary file 2 — Table S1‐S3 [file MGG3-9-e1571-s002.docx]

Table S1. Mutagenic primers

| Variants | Sequence (5’→3’) |
| --- | --- |
| c.271G>A | cctgctgcagcacccaaactAagattgtggaga |
| c.275T>C | tgctgcagcacccaaactgagaCtgtggagaccttg |
| c.631G>A | attcactttaaagcccagtggAcaaatccttttgatc |
| c.880C>T | aaaacactgaagaagtggaactgcttactaca |
| c.909G>T | gaagggatgggttgacttTtttgttccaaag |
| c.927del | tgtttgttccaaagttttcatttctgccacatatgacc |

Table S2 misdiagnose and error therapy of probands from family E

| Patients | Times | Levothyroxine (μg) | TT3 (nmol/L) | TT4 (nmol/L) | FT3 (pmol/L) | FT4 (pmol/L) | TSH (mU/L) | TBG (μg/mL) |
| --- | --- | --- | --- | --- | --- | --- | --- | --- |
| II-1  (Family E) | Initial diagnosis | 75 | 1.40 | **36.0↓** | ND | ND | 1.45 | ND |
|  | Eight months later | 125 | 1.90 | 64.2 | 6.00 | 16.10 | **0.00↓** | ND |
|  | Dosage reduction | 50 | 1.42 | **53.1↓** | **6.92↑** | 17.03 | **0.01↓** | **11.0↓** |
|  | Drug withdrawal | 0 | 1.27 | **41.5↓** | 5.64 | 17.27 | **1.31↓** | ND |
| II-2  (Family E) | Initial diagnosis | 50 | 1.20 | **40.1↓** | ND | ND | 1.01 | ND |
|  | Eight months later | 100 | 1.70 | 65.8 | **6.88↑** | 20.97 | **0.00↓** | ND |
|  | Dosage reduction | 50 | **0.90↓** | **52.4↓** | 5.60 | 22.90 | **0.02↓** | **10.4↓** |
|  | Drug withdrawal | 0 | 1.12 | **35.4↓** | 5.69 | 15.41 | 0.96 | ND |

TT3: total triiodothyronine (1.02-2.96 nmol/L); TT4: total thyroxine (55.4-161.3 nmol/L); FT3: free triiodothyronine (2.77-6.31 pmol/L); FT4: free thyroxine (10.5-24.4 pmol/L); TSH: thyroid stimulating hormone (0.38-4.34 mIU/L); TBG: thyroxine-binding globulin (13.0-39.0 μg/mL); ND: Not done; The abnormal values are in bold.

Table S3 Serum TBG levels in individuals with p.L303F hemizygotes or p.L303F homozygotes and wild-type genotype controls.

| Family members | Sex | Variants in TBG | TT4  nmol/L | TBG  (μg/mL) | References |
| --- | --- | --- | --- | --- | --- |
| II-1 | Female | p.L303F Homozygotes | 86.2 | 17.30 | 8 |
| II-4 | Male | p.L303F Hemizygotes | 81.1 | 17.20 |  |
| III-1 | Male | p.L303F Hemizygotes | 84.8 | 17.60 |  |
| III-2 | Male | p.L303F Hemizygotes | 73.3 | 10.70 |  |
| III-4 | Male | p.L303F Hemizygotes | 75.0 | 15.30 |  |
| III-5 | Male | p.L303F Hemizygotes | 86.3 | 10.40 |  |
| IV-8 | Male | p.L303F Hemizygotes | 71.1 | 8.90 |  |
| IV-9 | Female | p.L303F Homozygotes | 90.4 | 13.60 |  |
| II-2 | Male | p.L303F Hemizygotes | 62.09 | 9.6 | 9 |
| I-1 (A) | Male | p.L303F Hemizygotes | 52.4 | 8.8 | Present study |
| II-1 (A) | Male | p.L303F Hemizygotes | 53.4 | 9.8 |  |
| II-1 (B) | Male | p.L303F Hemizygotes | 62.8 | 10.1 |  |
| I-1 (C) | Male | p.L303F Hemizygotes | 55.8 | 14.6 |  |
| I-1 (E) | Male | p.L303F Hemizygotes | 91.1 | 13.8 |  |
| II-3 (E) | Male | p.L303F Hemizygotes | 100.8 | 13.0 |  |
| I-1 (F) | Male | p.L303F Hemizygotes | 55.6 | 12.2 |  |
| I-1 (I) | Male | p.L303F Hemizygotes | 53.1 | 14.5 |  |
| II-2 | Male | Wild-type | 132.7 | 21.30 | 8 |
| II-5 | Female | Wild-type | 107.6 | 23.50 |  |
| II-8 | Male | Wild-type | 113.6 | 33.00 |  |
| III-9 | Male | Wild-type | 94.5 | 20.90 |  |
| IV-2 | Male | Wild-type | 104.8 | 21.50 |  |
| IV-6 | Male | Wild-type | 114.3 | 21.80 |  |
| IV-10 | Male | Wild-type | 99.9 | 16.40 |  |
| IV-12 | Male | Wild-type | 93.8 | 18.70 |  |
| IV-13 | Male | Wild-type | 116.9 | 23.00 |  |
| IV-14 | Female | Wild-type | 103.9 | 21.50 |  |
| I-2 | Male | Wild-type | 90.96 | 17.8 | 9 |
| AII/1 | Male | Wild-type | - | 30.0 | 6 |
| I-I (B) | Male | Wild-type | 73.9 | 16.5 | Present study |
| I-1 (D) | Male | Wild-type | 67.5 | 25.3 |  |
| I-1 (H) | Male | Wild-type | 67.9 | 21.9 |  |
| I-1 (J) | Male | Wild-type | 70.8 | 22.3 |  |
